# Immunological Impacts of Diabetes on the Susceptibility of Mycobacterium tuberculosis

**DOI:** 10.1155/2019/6196532

**Published:** 2019-09-09

**Authors:** Birhanu Ayelign, Markos Negash, Meaza Genetu, Tadelo Wondmagegn, Tewodros Shibabaw

**Affiliations:** ^1^Department of Immunology and Molecular Biology, School of Biomedical and Laboratory Science, College of Medicine and Health Sciences, University of Gondar, Ethiopia; ^2^Department of Biochemistry, College of Medicine and Health Sciences, University of Gondar, Ethiopia

## Abstract

The interaction between diabetes and major world infections like TB is a major public health concern because of rapidly rising levels of diabetes. The dual burden of tuberculosis (TB) and diabetes mellitus (DM) has become a major global public health problem. Diabetes mellitus is a major risk factor for the development of active and latent tuberculosis. Immune mechanisms contributing to the increased susceptibility of diabetic patients to *TB* are due to the defects in bacterial recognition, phagocytic activity, and cellular activation which results in impaired production of chemokines and cytokines. The initiation of adaptive immunity is delayed by impaired antigen-presenting cell (APC) recruitment and function in hyperglycemic host, which results in reduced frequencies of Th1, Th2, and Th17 cells and its secretion of cytokines having a great role in activation of macrophage and inflammatory response of tuberculosis. In addition, impaired immune response and killing of intracellular bacteria potentially increase bacterial load, chronic inflammation, and central necrosis that facilitate bacterial dissemination and miliary tuberculosis. Understanding of the immunological and biochemical basis of *TB* susceptibility in diabetic patients will tell us the rational development of implementation and therapeutic strategies to alleviate the dual burden of the diseases. Therefore, the aim of this review was focused on the association between diabetes and tuberculosis, focusing on epidemiology, pathogenesis, and immune dysfunction in diabetes mellitus, and its association with susceptibility, severity, and treatment outcome failure to tuberculosis.

## 1. Background

### 1.1. Epidemiology and Pathogenesis of Diabetes Mellitus

Diabetes mellitus is a metabolic disorder characterized by chronic hyperglycemia resulting from defect in insulin resistance or/and secretion [1, 2]. Globally, 422 million peoples were living with diabetes in 2014, and over the past decade, diabetes prevalence has raised faster in low- and middle-income countries [2]. Approximately 90 to 95% of the diagnosed diabetic population has type 2 diabetes [3, 4]. Type 1 diabetes mellitus is an autoimmune disease associated with destruction of insulin-producing pancreatic *β*-cells [5, 6]. It results from the formation of specific self-islet *β* antigen, and these autoantigens are presented by antigen-presenting cells to activate T helper (Th1 and Th2) cells [7, 8]. Activated Th1 cell produces interleukin-2 (IL-2) to activate T cytotoxic cell which destroys the islet cells through the secretion of toxic chemicals perforins and granzymes, and interferon gamma (IFN*γ*) activates macrophages and stimulates the release of inflammatory cytokines like IL-1 and tumor necrosis factor alpha (TNF*α*) which further destroy beta cells [8–10]. The CD4+ T cells can also activate islet antigen-specific B cells to produce antibodies that mediate complement killing as well as binding to Fc receptors on macrophages [8, 10, 11]. T2DM results from impaired insulin secretion and increased insulin resistance, affected by genetic and environmental factors including obesity [12]. Obesity induces adiposity hypertrophy and changes in stromovascular cell composition to bust the proinflammatory state which leads to interaction of adaptive cells with adipose tissue macrophages to modify their activation state [13]. In obesity and type 2 diabetes, adipose tissue is characterized by macrophages and T lymphocytes with a shift from an anti-inflammatory to a proinflammatory state [13, 14]. CD8+ and CD4+ Th1 and Th17 cells stimulate classical macrophage polarization; additionally, B lymphocytes and mast cells also increase in obese adipose tissue contributing to obesity-induced inflammation. Despite this fact, the imbalance between immune cells results in the production of excess chemokines and proinflammatory cytokines that promote systemic inflammation by serine phosphorylation resulting in peripheral insulin resistance via inhibition of tyrosine phosphorylation [14–16]. Subsequently, this immunological dysfunction leads to diabetic patients being more risky toward many infectious diseases. Diabetes is a common predisposing factor to severity of respiratory infection (*Streptococcus pneumoniae*, *Staphylococcus aureus*, *Haemophilus influenzae*, other gram-negative bacilli, atypical pathogen, and tuberculosis), urinary tract infections (*Escherichia coli*, Proteus species, and other gram-negative bacilli), and soft tissue infections including foot [17, 18] and fungal infections in oropharyngeal, vulvovaginitis, and cutaneous candidiasis [17, 19]. The study suggested that diabetic patients more likely had high bacterial loads of Staphylococcus aureus than nondiabetics to have endocarditis; however, there had no increase in mortality [20]. In addition, some chronic diseases are more prevalent in diabetic persons, like malignant external otitis, rhinocerebral mucormycosis, and gangrenous cholecystitis [17, 18]. Thus, the main pathogenic mechanisms on the way to the risk of diabetic patients to infections are a hyperglycemic environment that increases the virulence of pathogens by a lower production of interleukins in response to infection, reduced chemotaxis and phagocytic activity, immobilization of polymorphonuclear leukocytes, and glycosuria and gastrointestinal and urinary dysmotility [18, 21].

## 2. Effects and Immunological Mechanism of Diabetes on Tuberculosis Susceptibility

A person who has diabetes mellitus are approximately 3 times more likely to develop tuberculosis than nondiabetics [22]. Tuberculosis (TB) is an infectious disease caused by the intercellular Mycobacterium tuberculosis (MTB) [23]. Many risk factors contribute to LTBI and TB disease at both individual and population levels [24]. Thus, DM is one of the chronic diseases that are risk factors for the conversion of latent to active tuberculosis [25]. Scholars hypothesized that an impaired immune function due to dysglycemia in both diabetic and prediabetic patients is more likely to have LTBI than those without DM [26].

Globally, there are an estimated 9.6 million new patients with active TB annually; from those, one million people have both TB and DM [27]. Despite this rising prevalence, DM is a potential challenge to control and treat TB [28]. The World Health Organization (WHO) has recommended important intervention strategies to reduce the dual burden of TB-DM comorbidity, namely, establishing mechanisms of collaboration between TB and DM control programs, detection and management of TB in patients with DM, and detection and management of DM in TB patients [29]. However, for this strategy, it is crucial to understand the magnitude and immunological mechanisms of TB-DM comorbidity, particularly in low- and middle-income countries. Despite this, we review the prevalence of diabetes-tuberculosis coinfection in different countries. It shows that there was a high prevalence in India (29%), Korea (26.5%), and Mexico-Texas (25%) followed by Addis Ababa, Ethiopia (15.8%) (Table 1).

High susceptibility to infections, including TB, is a major cause of morbidity and mortality in patients with diabetes, and the probable cause of increased prevalence and complication of the infections in DM patients is immune dysfunction [40, 41]. Natural infection with *MTB* occurs by inhalation of bacilli that invade and replicate in alveolar macrophages and horizontally spread to macrophages, myeloid DCs, and neutrophils recruited from the periphery, which results in priming of adaptive immunity [42, 43]. In M. tuberculosis infection, complement has also a great role to promote the opsonization and phagocytosis of microorganisms through macrophages and neutrophils and induce the lysis of these microorganisms [18, 44]. Complement component C3 enhances the adherence and uptake of *M. tuberculosis* by mononuclear phagocytes [45, 46]. Moreover, complement activation products provide the second signal for B-lymphocyte activation and antibody production [45].

**Figure 1:**
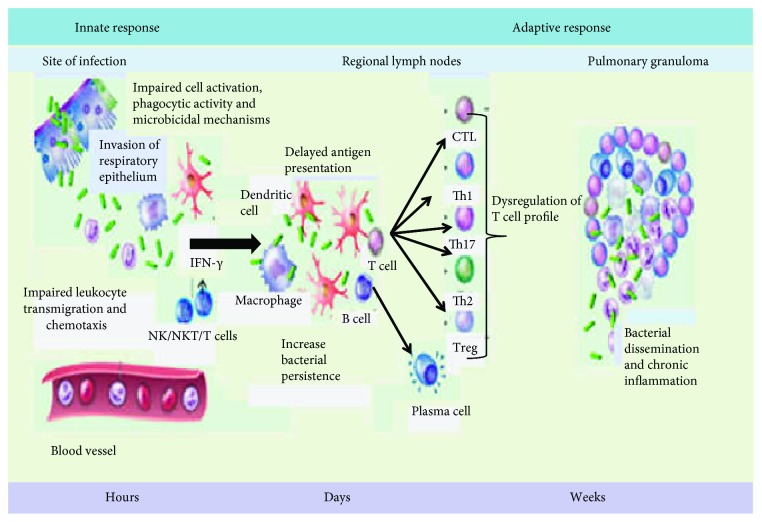
The putative immune mechanisms contributing to the increased susceptibility of diabetic hosts to Mycobacterium tuberculosis.

Despite this, antigen-specific T cells expand and travel to the lung where they promote an effective antimicrobial response as a result of activating macrophage through release of IFN*γ* and cytotoxic T cell targeting of *MTB*-infected macrophages [21]. But few studies indicated that TB susceptibility has also been reported in rat models of type 1 and type 2 DM [41, 43]. However, the immunological mechanisms of susceptibility to TB among those with DM still needed clear understanding. Increased susceptibility to TB in patients with DM has been endorsed to several factors, including direct effects related to hyperglycemia and insulin resistance and indirect effects related to macrophage and lymphocyte function [47]. The impaired immune response in patients with DM facilitates either primary infection with TB or reactivation of latent TB, may be possible for these defective immune responses (Figure 1). Therefore, below, studies point out the innate and adaptive immune responses to *MTB* antigen in patients with DM.

### 2.1. Innate Immune Dysfunction in DM Patients and Susceptibility to TB

Differences in innate immunity between diabetic and nondiabetic patients are more significant in the susceptibility and pathogenesis of infections including TB [42, 48]. It has been revealed that the function of neutrophils, macrophages, DC, NK cells, and some other components of innate immunity is drastically compromised by metabolic alterations in DM [40, 42, 49]. Thus, immune dysfunction may play an important role in the reactivation and host's susceptibility to exogenous infection of TB [49]. The initial infection of alveolar macrophages (AM) by inhaled *MTB* activates an innate response that recruits multiple myeloid cell types to the alveolar airspace [50]. Alveolar macrophages have a central role in hosts for *Mycobacterium tuberculosis* infection and replication [48]. These macrophages ingest the bacilli to enclose them in phagosomes and fuse with lysosome along with digestion of the bacteria and production of antimicrobial molecules like reactive nitrogen intermediates [21]. It has also an essential role in the formation of hallmark feature of tuberculosis in humans, the so-called “granulomas,” which contain other immune effector cells, such as neutrophils and T cell [21, 51]. Alveolar macrophages from diabetic mice had increased the expression of CCR2, which may restrain monocyte traffic to the lung, and reduced expression of CD14 and macrophage receptor which recognizes the *MTB* cell wall components that contribute reduced phagocytosis of *MTB* and increase tuberculosis susceptibility in diabetic hosts [50, 52].

An experimental study by aerosol challenging hyperglycemic mice and euglycemic control mice indicated that the function of *MTB*-infected AM is impaired in hyperglycemic mice resulting in a reduced expression of signals and chemokines that recruit macrophages, DCs, neutrophils, and innate lymphocytes to the airspace, and also, it produces a barrier to leukocyte transmigration into the airspace even if adequate recruiting signals are produced by infected AM [42, 50]. In addition, IFN*γ* also determines the activation of macrophage, on which its production is mediated by the release of IL-1*β*, IL-12, and IL-18 from APCs upon stimulation with mycobacteria. Therefore, type 2 diabetes patients might be characterized by decreased secretion of IL-1*β*, IL-12, and IL-18 and respond with less IFN*γ* upon stimulation, leading to increased susceptibility to TB [53]. Dendritic cells [54] are one of the APCs which link both innate and adaptive immune cells [47]. The migration of DC to the draining lymph node is essential for the activation of naive T cells in TB infection [47, 55, 56]. Studies revealed that TB-DM individuals showed significantly lower frequencies of both myeloid DC and plasmacytoid DC compared with individuals with TB; however, the contribution of the pathogenesis was not clearly understood. Therefore, they bring to a close; hyperglycemia is the primary influence in the alterations of DC frequency in TB-DM [43, 47, 56]. Neutrophils are the first cell that migrates to infected tissue to kill the bacteria and secrete a wide range of cytokines and chemokines which induce other immune cell recruitment and activations [43, 48]. Multiple receptors including TLRs, C-type lectin receptors (CLRs), and cytokine receptors have been implicated in the interaction between neutrophils, M. tuberculosis, and proinflammatory cytokines [57]. Moreover, neutrophils have a great implication in the acute inflammatory response to M. tuberculosis [58]. The impact of hyperglycemia on neutrophils in TB has been investigated that increased adhesion and integrin expression, reduced chemotaxis, defected phagocytes, and reduced microbicidal activity as compared with neutrophils from euglycemic controls [43] (Figure 1). There was also evidence on which glycated collagen impedes neutrophil migration compared with nonglycated collagen due to the receptor for advanced glycation end products (RAGE), which is expressed on neutrophils and other leukocytes [42, 48].

### 2.2. Adaptive Immune Dysfunction in DM Patients and Susceptibility to TB

Adaptive immunity against *MTB* infection is mostly cellular immune responses [59]. T helper 1 (Th1) cells play a central role in the host defense by inducing the production of IFN*γ*, which potentiates the nitric oxide- (NO-) dependent killing activity of macrophages, while IL-2 is an essential cytokine for the development and proliferation of Th1 and CD8+ T cells, and Th17 cell secretes IL-17 and IL-23 that plays inflammatory response of TB [41, 42, 47, 53, 59]. They postulated that impaired Th cell function in DM would be a major factor for the development of TB [59]. Despite that, an increasing number of immunological studies in patients with DM who develop TB show an absurd hyperinflammatory response [47]. Several studies revealing that cytokine response of TB with DM versus without DM following in vitro stimulation of immune cells with purified mycobacterial antigens indicate that the secretion of IFN*γ* has been evaluated; however, results are contraindicated with studies showing either no difference [53] or lower [60] or higher [61, 62], and the difference may be due to types of stimulating antigen and pool of MTB region of difference. Another experimental study showed that the frequencies of functional Th1 cells are decreased in DM individuals compared to NDM individuals with TB infection [48, 55, 59, 60]. Diabetes mellitus might potentially influence and/or decrease the frequencies of Th1 and Th17 cells in TB-DM individuals due to increased frequencies of Th2 cells which secrete IL 4; as a result, Th2 cells are known to antagonize the differentiation of Th1 and Th17 cells [47, 59]. Irrespective of its cytokine secretion, another study indicates that the frequency of CD4+ T cells expressing Th2 cytokines is actually decreased in TB-DM individuals, suggesting that DM is related to alteration of antigen-specific frequencies of most CD4+ T cell subsets [55]. Since Th1 and Th2 cytokines regulate the secretions with each other, they found that the overall Th1/Th2 cytokine ratios (IFN*γ* : IL-4, IFN*γ* : IL-5, and IFN*γ* : IL-10 and TNF*β* : IL-4, TNF*β* : IL-5, and TNF*β* : IL-10) were lower in the diabetic TB patients than NDM-TB patients and healthy subjects. Consequently, lower Th1 : Th2 ratios may possibly contribute to susceptibility of MTB infection under diabetic conditions [61]. Another mechanism that could contribute to the diminished Th1, Th2, and Th17 response is due to increased frequencies of regulatory T cells in TB-DM disease. Regulatory T cell frequencies are significantly higher in DM compared to NDM individuals, on which IL-10 and TGF*β* are regulatory cytokines with a broad spectrum of activity, predominantly anti-inflammatory cytokine which interferes mycobacterial antigen-specific Th1 and Th2 cytokine production [61]. Even though there is production of IFN*γ* secretion, IL-10 can help mycobacteria to survive intracellularly and the elevated secretion of IL-10 may contribute to increased pathogenesis in diabetic TB patients [55, 59, 61] (Figure 1).

Figure 1 shows the putative immune mechanisms contributing to the increased susceptibility of diabetic hosts to Mycobacterium tuberculosis. Impaired cell activation and phagocytic activity lead to impaired production of chemokines and cytokines in diabetic hosts. Altered activation of natural killer (NK) cells, an early source of interferon *γ* (IFN*γ*) to enhance macrophage microbicidal activity, also facilitates intracellular bacterial persistence. Adaptive immunity is delayed by an impaired antigen-presenting cell (APC), and reduced frequency in diabetic hosts and dysregulation of the cytokine profile alter the activation and differentiation of T cell subsets. B cell activation and antibody production may also be impaired. The dysregulated inflammatory milieu affects granuloma formation, contributing to increased neutrophil recruitment and central necrosis that facilitates bacterial escape (Hodgson et al., 2015).

In humoral immune response, the role of antibody (Ab) either in pathogenesis or in protection against tuberculosis was controversial [63, 64]. Most studies indicate that the class of Abs are markers of disease progression and protection [64–66]. Furthermore, most scholars suggested that mechanisms of antibody-mediated protection against Mycobacterium tuberculosis are opsonization, increase of macrophage Ca^2+^ signaling and release of oxidants enhancing the intracellular killing, other mechanisms enhancing cell-mediated immunity, clearance of immunomodulatory mycobacterial antigens, direct antimycobacterial activity, and activation of complement [65, 67, 68]. In addition to this, hence, glycation of immunoglobulin and increased HbA1c in diabetes patients lose the biological function of the antibody [19].

### 2.3. Immunological Mechanism of Diabetes on the Severity and Treatment Outcome of Tuberculosis

*Mycobacterium tuberculosis* infection with diabetes is rapidly progressive with a shortened survival interval, more severe pulmonary and extrapulmonary pathologies, and a higher bacterial burden as compared to NDM controls [69]. Moreover, increase in disease severity, miliary TB, and higher bacterial load will contribute to increased mortality rates of TB-DM patients [69, 70]. Laboratory animal-based studies indicate that diabetes induction increases the frequency of airway shedding of M. tuberculosis even in the absence of cavitations, which may be related to a higher pulmonary bacterial load and/or an alteration in the diabetic airway microenvironment [69]. In chronic stages of hyperglycemia guinea pigs, there were higher expressions of TNF*α* and IL-1*β* and extrapulmonary bacterial burden than in NDM controls which lead to increasing the severity and disease progression of TB [55]. Therefore, the severity of the disease may be due to the altered expression of particular cytokines and subsequent cellular immune response to *MTB* infection [27, 48, 71]. High levels of IL-17 and IL-8 in diabetes with TB may be related to more granulocytic infiltration and pathology, and these byproducts of chronic hyperglycemia, combined with oxidative stress, induce a proinflammatory response which contributes to more severe inflammation and TB disease with type 2 diabetes [49, 71]. In general, Th1-based cytokine secretion is critical in the activation of macrophages for the protection of *MTB* infection. Despite this, M. tuberculosis infection with a diabetic is failing to control bacterial growth due to increased anti-inflammatory cytokine levels and IL-4 which inhibits the expression of IFN*γ*, resulting in rapid disease progression before the onset of adaptive immunity to M. tuberculosis in diabetic patients [69, 71]. Patients with diabetes are also more likely to fail treatment and die during treatment compared to those without diabetes [72–74]. A cohort study has shown that diabetes was independently associated with an increased risk of death and late culture conversion in patients undergoing treatment of TB compared with patients without diabetes [75, 76]. The possible hypothesis of delay in time of clearance and treatment failure of TB among DM patients is related to higher bacterial burden at diagnosis, which could be related to slower kinetics in the immune response in DM patients and altered pharmacokinetics of anti-TB drugs in TB patients with DM (absorption, distribution, metabolism, and excretion of drugs) [73, 76–78].

In conclusion, tuberculosis is one of the major causes of morbidity and mortality among infectious diseases worldwide, including Ethiopia. Diabetic patients induce an abnormal function in both innate and adaptive immune responses, which increased risk of the combined TB disease development, complication, outcomes of treatment failure, and death. The immunological mechanisms of diabetes to susceptibility of tuberculosis are complex and not fully elucidated. Research suggests that immune dysfunction in diabetes is more susceptible to tuberculosis. However, results from studies so far remain inconsistent. Therefore, further studies are required to fully understand the impact of DM patients on the immune response and increased susceptibility and treatment outcome of TB.

## Figures and Tables

**Table 1:** Summary of 10 studies of the association between diabetes and tuberculosis, at the University of Gondar, Ethiopia, 2018.

Study design	Country	Study period	TB-DM prevalence	Reference
Prospective	Mexico & Texas	March 2006 & September 2008	25%	[30]
Cross-sectional	India	—	29%	[31]
Cross-sectional	India	December 2014-June 2015	10.6%	[32]
Retrospective	Korea	1988-1990	26.5%	[33]
Retrospective	China	2008-2009	9.48%	[34]
Cohort	Taiwan	2002-2011	4.3-fold	[35]
Cross-sectional	Pakistan	September2014-August 2015	14.8%	[36]
Cross-sectional	Nigeria		5.7%	[37]
Cross-sectional	Addis Ababa, Ethiopia	February-May 2014	15.8%	[38]
Cross-sectional	Dessie, Ethiopia	February-April 2012	6.2%	[39]

## Additional Points

*Supporting Information*. PRISMA 2009 Checklist was used for impact of diabetes mellitus on susceptibility to *Mycobacterium tuberculosis* (DOC).

## References

[1] World Health Organization (1999). *Definition, diagnosis and classification of diabetes mellitus and its complications: report of a WHO consultation. Part 1: diagnosis and classification of diabetes mellitus*.

[2] WHO (2016). *Global Report on Diabetes*.

[3] Badawi A., Klip A., Haddad P. (2010). Type 2 diabetes mellitus and inflammation: prospects for biomarkers of risk and nutritional intervention. *Diabetes, Metabolic Syndrome and Obesity: Targets and Therapy*.

[4] Whiting D. R., Guariguata L., Weil C., Shaw J. (2011). IDF diabetes atlas: global estimates of the prevalence of diabetes for 2011 and 2030. *Diabetes Research and Clinical Practice*.

[5] Bouvy M. (1997). Applied therapeutics: the clinical use of drugs. *Pharmacy World and Science*.

[6] Ozougwu J. C., Obimba K. C., Belonwu C. D., Unakalamba C. B. (2013). The pathogenesis and pathophysiology of type 1 and type 2 diabetes mellitus. *Journal of Physiology and Pathophysiology*.

[7] Eisenbarth G. S. (2005). Type 1 diabetes mellitus. *Joslin’s Diabetes Mellitus*.

[8] Narendran P., Estella E., Fourlanos S. (2005). Immunology of type 1 diabetes. *QJM: An International Journal of Medicine*.

[9] Rossini A. A., Mordes J. P., Like A. A. (1985). Immunology of insulin-dependent diabetes mellitus. *Annual Review of Immunology*.

[10] Yagi H., Matsumoto M., Kunimoto K., Kawaguchi J., Makino S., Harada M. (1992). Analysis of the roles of CD4+ and CD8+ T cells in autoimmune diabetes of NOD mice using transfer to NOD athymic nude mice. *European Journal of Immunology*.

[11] Wållberg M., Cooke A. (2013). Immune mechanisms in type 1 diabetes. *Trends in Immunology*.

[12] Frostegård J. (2013). Immune mechanisms in atherosclerosis, especially in diabetes type 2. *Frontiers in Endocrinology*.

[13] Esser N., Legrand-Poels S., Piette J., Scheen A. J., Paquot N. (2014). Inflammation as a link between obesity, metabolic syndrome and type 2 diabetes. *Diabetes Research and Clinical Practice*.

[14] Cruz N. G., Sousa L. P., Sousa M. O., Pietrani N. T., Fernandes A. P., Gomes K. B. (2013). The linkage between inflammation and type 2 diabetes mellitus. *Diabetes Research and Clinical Practice*.

[15] Mohamed H. G., Idris S. B., Ahmed M. F. (2015). Influence of type 2 diabetes on local production of inflammatory molecules in adults with and without chronic periodontitis: a cross-sectional study. *BMC Oral Health*.

[16] Navarro-González J. F., Mora-Fernández C. (2008). The role of inflammatory cytokines in diabetic nephropathy. *Journal of the American Society of Nephrology*.

[17] Joshi N., Caputo G. M., Weitekamp M. R., Karchmer A. W. (1999). Infections in patients with diabetes mellitus. *New England Journal of Medicine*.

[18] Casqueiro J., Casqueiro J., Alves C. (2012). Infections in patients with diabetes mellitus: a review of pathogenesis. *Indian Journal of Endocrinology and Metabolism*.

[19] Peleg A. Y., Weerarathna T., McCarthy J. S., Davis T. M. E. (2007). Common infections in diabetes: pathogenesis, management and relationship to glycaemic control. *Diabetes/Metabolism Research and Reviews*.

[20] Cooper G., Platt R. (1982). Staphylococcus aureus bacteremia in diabetic patients: endocarditis and mortality. *The American Journal of Medicine*.

[21] Palaga T. (2017). An update on the immunology of tuberculosis. *Siriraj Medical Journal*.

[22] Jeon C. Y., Murray M. B. (2008). Diabetes mellitus increases the risk of active tuberculosis: a systematic review of 13 observational studies. *PLoS Medicine*.

[23] World Health Organization (2016). Global tuberculosis report 2016.

[24] Remy W. L. (2016). The association between latent tuberculosis infection and diabetes mellitus control in the United States. *Theses and Dissertations--Public Health*.

[25] Hartman-Adams H., Clark K., Juckett G. (2014). Update on latent tuberculosis infection. *American Family Physician*.

[26] Hensel R. L., Kempker R. R., Tapia J., Oladele A., Blumberg H. M., Magee M. J. (2016). Increased risk of latent tuberculous infection among persons with pre-diabetes and diabetes mellitus. *The International Journal of Tuberculosis and Lung Disease*.

[27] Lönnroth K., Roglic G., Harries A. D. (2014). Improving tuberculosis prevention and care through addressing the global diabetes epidemic: from evidence to policy and practice. *The Lancet Diabetes & Endocrinology*.

[28] Workneh M. H., Bjune G. A., Yimer S. A. (2017). Prevalence and associated factors of tuberculosis and diabetes mellitus comorbidity: a systematic review. *PLoS One*.

[29] WHO (2013). *Collaborative Framework for Care and Control of Tuberculosis and Diabetes*.

[30] Restrepo B. I., Camerlin A. J., Rahbar M. H. (2011). Cross-sectional assessment reveals high diabetes prevalence among newly-diagnosed tuberculosis cases. *Bulletin of the World Health Organization*.

[31] Raghuraman S., Vasudevan K. P., Govindarajan S., Chinnakali P., Panigrah K. C. (2014). Prevalence of diabetes mellitus among tuberculosis patients in urban Puducherry. *North American Journal of Medical Sciences*.

[32] Marupuru S., Senapati P., Pathadka S., Miraj S. S., Unnikrishnan M. K., Manu M. K. (2017). Protective effect of metformin against tuberculosis infections in diabetic patients: an observational study of south Indian tertiary healthcare facility. *The Brazilian Journal of Infectious Diseases*.

[33] Kim S. J., Hong Y. P., Lew W. J., Yang S. C., Lee E. G. (1995). Incidence of pulmonary tuberculosis among diabetics. *Tubercle and Lung Disease*.

[34] Zhang Q., Xiao H., Sugawara I. (2009). Tuberculosis complicated by diabetes mellitus at Shanghai Pulmonary Hospital, China. *Japanese Journal of Infectious Diseases*.

[35] Shen T.-C., Lin C.-L., Wei C.-C. (2014). Increased risk of tuberculosis in patients with type 1 diabetes mellitus: results from a population-based cohort study in Taiwan. *Medicine*.

[36] Zahr R. S., Peterson R. A., Polgreen L. A. (2016). Diabetes as an increasingly common comorbidity among patient hospitalizations for tuberculosis in the USA. *BMJ Open Diabetes Research and Care*.

[37] Olayinka A. O., Anthonia O., Yetunde K. (2013). Prevalence of diabetes mellitus in persons with tuberculosis in a tertiary health centre in Lagos, Nigeria. *Indian Journal of Endocrinology and Metabolism*.

[38] Damtew E., Ali I., Meressa D. (2014). Prevalence of diabetes mellitus among active pulmonary tuberculosis patients at St. Peter Specialized Hospital, Addis Ababa, Ethiopia. *World Journal of Medical Sciences*.

[39] Amare H., Gelaw A., Anagaw B., Gelaw B. (2013). Smear positive pulmonary tuberculosis among diabetic patients at the Dessie referral hospital, Northeast Ethiopia. *Infectious Diseases of Poverty*.

[40] Geerlings S. E., Hoepelman A. I. M. (1999). Immune dysfunction in patients with diabetes mellitus (DM). *FEMS Immunology & Medical Microbiology*.

[41] Martens G. W., Arikan M. C., Lee J., Ren F., Greiner D., Kornfeld H. (2007). Tuberculosis susceptibility of diabetic mice. *American Journal of Respiratory Cell and Molecular Biology*.

[42] Martinez N., Kornfeld H. (2014). Diabetes and immunity to tuberculosis. *European Journal of Immunology*.

[43] Raposo-García S., Guerra-Laso J. M., García-García S. (2017). Immunological response to Mycobacterium tuberculosis infection in blood from type 2 diabetes patients. *Immunology Letters*.

[44] Owen J. A., Punt J., Stranford S. A. (2013). *Kuby Immunology*.

[45] Manivannan S., Rao N. V., Ramanathan V. D. (2012). Role of complement activation and antibody in the interaction between *Mycobacterium tuberculosis* and human macrophages. *Indian Journal of Experimental Biology*.

[46] Schlesinger L., Bellinger-Kawahara C. G., Payne N. R., Horwitz M. A. (1990). Phagocytosis of Mycobacterium tuberculosis is mediated by human monocyte complement receptors and complement component C3. *The Journal of Immunology*.

[47] Nathella P. K., Babu S. (2017). Influence of diabetes mellitus on immunity to human tuberculosis. *Immunology*.

[48] Restrepo B. I., Schlesinger L. S. (2013). Host-pathogen interactions in tuberculosis patients with type 2 diabetes mellitus. *Tuberculosis*.

[49] Yew W. W., Leung C. C., Zhang Y. (2017). Oxidative stress and TB outcomes in patients with diabetes mellitus?. *Journal of Antimicrobial Chemotherapy*.

[50] Martinez N., Ketheesan N., West K., Vallerskog T., Kornfeld H. (2016). Impaired recognition of Mycobacterium tuberculosis by alveolar macrophages from diabetic mice. *The Journal of Infectious Diseases*.

[51] Bozzano F., Marras F., de Maria A. (2014). Immunology of tuberculosis. *Mediterranean Journal of Hematology and Infectious Diseases*.

[52] Gomez D. I., Twahirwa M., Schlesinger L. S., Restrepo B. I. (2013). Reduced Mycobacterium tuberculosis association with monocytes from diabetes patients that have poor glucose control. *Tuberculosis*.

[53] Stalenhoef J. E., Alisjahbana B., Nelwan E. J. (2008). The role of interferon-gamma in the increased tuberculosis risk in type 2 diabetes mellitus. *European Journal of Clinical Microbiology & Infectious Diseases*.

[54] Lee D.-H., Ha M. H., Kim J. H. (2003). Gamma-glutamyltransferase and diabetes—a 4 year follow-up study. *Diabetologia*.

[55] Kumar N. P., Moideen K., George P. J., Dolla C., Kumaran P., Babu S. (2016). Coincident diabetes mellitus modulates Th1‐, Th2‐, and Th17‐cell responses in latent tuberculosis in an IL‐10‐ and TGF‐*β*‐dependent manner. *European Journal of Immunology*.

[56] Kumar N. P., Moideen K., Sivakumar S. (2016). Modulation of dendritic cell and monocyte subsets in tuberculosis-diabetes co-morbidity upon standard tuberculosis treatment. *Tuberculosis*.

[57] Dallenga T., Linnemann L., Paudyal B., Repnik U., Griffiths G., Schaible U. E. (2018). Targeting neutrophils for host-directed therapy to treat tuberculosis. *International Journal of Medical Microbiology*.

[58] Kroon E. E., Coussens A. K., Kinnear C. (2018). Neutrophils: innate effectors of TB resistance?. *Frontiers in Immunology*.

[59] Yamashiro S., Kawakami K., Uezu K. (2005). Lower expression of Th1-related cytokines and inducible nitric oxide synthase in mice with streptozotocin-induced diabetes mellitus infected with *Mycobacterium tuberculosis*. *Clinical & Experimental Immunology*.

[60] Meenakshi P., Ramya S., Lavanya J., Vijayalakshmi V., Sumanlatha G. (2016). Effect of IFN-*γ*, IL-12 and IL-10 cytokine production and mRNA expression in tuberculosis patients with diabetes mellitus and their household contacts. *Cytokine*.

[61] Al‐Attiyah R. J., Mustafa A. S. (2009). Mycobacterial antigen-induced T helper type 1 (Th1) and Th2 reactivity of peripheral blood mononuclear cells from diabetic and non-diabetic tuberculosis patients and *Mycobacterium bovis* bacilli Calmette–Guérin (BCG)-vaccinated healthy subjects. *Clinical & Experimental Immunology*.

[62] Gan S. H., KhinMar K. W., Barkham T. M. (2014). Interferon-*γ* responses to *Mycobacterium tuberculosis*-specific antigens in diabetes mellitus. *The European Respiratory Journal*.

[63] Lu L. L., Chung A. W., Rosebrock T. R. (2016). A functional role for antibodies in tuberculosis. *Cell*.

[64] Mattos A. M. M., Chaves A. S., Franken K. L. M. C. (2016). Detection of IgG1 antibodies against *Mycobacterium tuberculosis* DosR and Rpf antigens in tuberculosis patients before and after chemotherapy. *Tuberculosis*.

[65] Zimmermann N., Thormann V., Hu B. (2016). Human isotype-dependent inhibitory antibody responses against Mycobacterium tuberculosis. *EMBO Molecular Medicine*.

[66] Feris E. J., Encinales L., Awad C. (2016). High levels of anti-tuberculin (IgG) antibodies correlate with the blocking of T-cell proliferation in individuals with high exposure to *Mycobacterium tuberculosis*. *International Journal of Infectious Diseases*.

[67] Achkar J. M., Chan J., Casadevall A. (2015). Role of B cells and antibodies in acquired immunity against Mycobacterium tuberculosis. *Cold Spring Harbor Perspectives in Medicine*.

[68] Jacobs A. J., Mongkolsapaya J., Screaton G. R., McShane H., Wilkinson R. J. (2016). Antibodies and tuberculosis. *Tuberculosis*.

[69] Podell B. K., Ackart D. F., Obregon-Henao A. (2014). Increased severity of tuberculosis in guinea pigs with type 2 diabetes: a model of diabetes-tuberculosis comorbidity. *The American Journal of Pathology*.

[70] Ruslami R., Aarnoutse R. E., Alisjahbana B., van der Ven A. J. A. M., van Crevel R. (2010). Implications of the global increase of diabetes for tuberculosis control and patient care. *Tropical Medicine & International Health*.

[71] Kumar N. P., Sridhar R., Banurekha V. V., Jawahar M. S., Nutman T. B., Babu S. (2013). Expansion of pathogen-specific T-helper 1 and T-helper 17 cells in pulmonary tuberculosis with coincident type 2 diabetes mellitus. *The Journal of Infectious Diseases*.

[72] Khalil N. H., Ramadan R. A. (2016). Study of risk factors for pulmonary tuberculosis among diabetes mellitus patients. *Egyptian Journal of Chest Diseases and Tuberculosis*.

[73] Suleiman S. A. S., Ishaq Aweis D. M., Mohamed A. J., RazakMuttalif A., Moussa M. A. A. (2012). Role of diabetes in the prognosis and therapeutic outcome of tuberculosis. *International Journal of Endocrinology*.

[74] Chiang C. Y., Bai K. J., Lin H. H. (2015). The influence of diabetes, glycemic control, and diabetes-related comorbidities on pulmonary tuberculosis. *PLoS One*.

[75] Dooley K. E., Golub J. E., Cronin W., Tang T., Dorman S. E. (2009). Impact of diabetes mellitus on treatment outcomes of patients with active tuberculosis. *The American Journal of Tropical Medicine and Hygiene*.

[76] Heysell S. K., Moore J. L., Staley D., Dodge D., Houpt E. R. (2013). Early therapeutic drug monitoring for isoniazid and rifampin among diabetics with newly diagnosed tuberculosis in Virginia, USA. *Tuberculosis Research and Treatment*.

[77] Restrepo B. I., McCormick J. B., Smith B., Jeon S., Rahbar M. H., Fisher-Hoch S. P. (2008). Mycobacterial clearance from sputum is delayed during the first phase of treatment in patients with diabetes. *The American Journal of Tropical Medicine and Hygiene*.

[78] Alisjahbana B., Sahiratmadja E., Nelwan E. J. (2007). The effect of type 2 diabetes mellitus on the presentation and treatment response of pulmonary tuberculosis. *Clinical Infectious Diseases*.

